# PREDICTORS OF FUNCTIONAL ABILITY VARY BY AGE

**DOI:** 10.1093/geroni/igad104.2878

**Published:** 2023-12-21

**Authors:** Carly Pullen, Julie Hicks Patrick

**Affiliations:** West Virginia University, Morgantown, West Virginia, United States; West Virginia University, Morgantown, West Virginia, United States

## Abstract

Across adulthood, chronic physical and emotional conditions can interfere with wellbeing. We examined the potential moderating effect of age on the process functional ability Using data from 401,958 adults who completed the 2020 Behavioral Risk Factor Surveillance System survey, we tested the associations among age, sex, race, chronic health conditions, poor mental health, and functional ability. The initial model was a poor fit to the data, χ 2 (DF = 24) = 592039, p > .001; RMSEA = .25. In order to examine whether this poor fit was related to age differences in the model, we removed age as a predictor and included it as a moderator. Thus, we tested the model among 122,005 younger adults, 140,122 midlife adults, and 139,831 older adults. The model fit improved, χ 2 (DF = 54) = 567119.79 p > .001; RMSEA = .25. Variance accounted for in functional ability differed, with 22% among younger adults, 33% at midlife, and 20.5% for older adults. Additional post hoc analyses were conducted and suggest alternate pathways to functional wellbeing across the lifespan. Our results are discussed the within the context of expanding investigations of functional wellbeing to include a set of predictors which captures both common and unique pathways across age.

